# Crystal structure and Hirshfeld analysis of 2-(5-bromo­thio­phen-2-yl)aceto­nitrile

**DOI:** 10.1107/S2056989018000968

**Published:** 2018-01-19

**Authors:** Ted M. Pappenfus, Tiana L. Wood, Joseph L. Morey, Wyatt D. Wilcox, Daron E. Janzen

**Affiliations:** a Division of Science and Mathematics, University of Minnesota, Morris, MN 56267, USA; b Dept. of Chemistry and Biochemistry, St. Catherine University, 20204 Randolph Avenue, St. Paul, MN 55105, USA

**Keywords:** crystal structure, thio­phene, nitrile, Hirshfeld analysis, halogen inter­actions

## Abstract

The title compound, C_6_H_4_BrNS, crystallizes in the space group *P*2_1_/*n* with one complete mol­ecule in the asymmetric unit. The non-H atoms are nearly planar (r.m.s for non-H atoms = 0.071 Å), with the nitrile group oriented anti­periplanar with respect to the thio­phene S atom. Inter­molecular Type I centrosymmetric Br⋯Br halogen inter­actions are present at a distance of 3.582 (1) Å and with a C—Br⋯Br angle of 140.7 (1)°. Additional weaker C—H⋯N, C—H⋯S, and S⋯π inter­actions are also present. A Hirshfeld analysis indicates Br⋯Br inter­actions comprise only 1.9% of all the inter­atomic contacts.

## Abstract 2

The crystal structure of 2-(5-bromo­thio­phen-2-yl)aceto­nitrile, previously reported as a liquid, has short centrosymmetric Type I Br⋯Br halogen inter­actions.

## Chemical context   

Cyano-substituted mol­ecules have found widespread use as functional materials for a variety of applications in organic electronics (Kim & Lim, 2014 ▸). For example, the title compound, 2-(5-bromo­thio­phen-2-yl)aceto­nitrile, has been incorporated into materials for use in organic semiconductors (Park *et al.*, 2016 ▸), sensors (Ding *et al.*, 2015 ▸), dye-sensitized solar cells (Li *et al.*, 2016 ▸), and organic solar cells (Kwon *et al.*, 2015 ▸). Although the chemical literature has previously identified the title compound, **1**, as a liquid (Cho *et al.*, 2004 ▸; Chung *et al.*, 2009 ▸; Lu *et al.*, 2014 ▸; Wan *et al.*, 2009 ▸; Zou *et al.*, 2009 ▸), we have found that with proper purification, this mol­ecule crystallizes under ambient conditions.
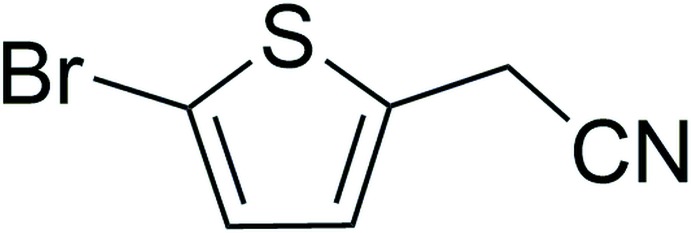


## Structural commentary   

**Figure 1:**
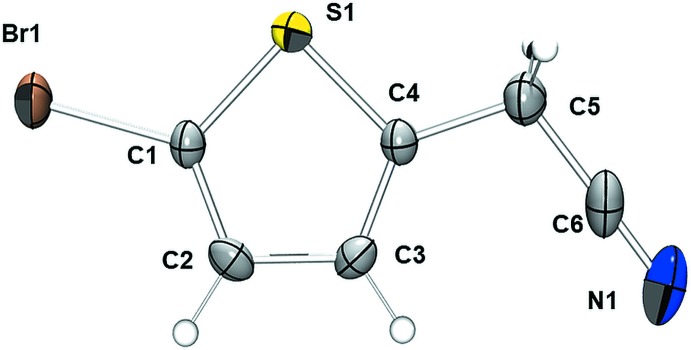
A displacement ellipsoid plot (50% probability ellipsoids for non-H atoms) of the asymmetric unit of **1**.

The mol­ecular structure of **1** is illustrated in Fig. 1 ▸. The asymmetric unit is composed of one complete mol­ecule of **1**. The C1—C2, C2—C3, and C3—C4 bond lengths are consistent with some conjugation in the thienyl π-system (Table 1 ▸). While both the C4—C5 and C5—C6 bond lengths are consistent with single C—C bonds, the C5—C6 bond length is shorter, likely as a result of the *sp* hybridization at C6. Although conjugation across the mol­ecule is not evident from the pattern of bond lengths, the structure is remarkably planar with an r.m.s. deviation from planarity of 0.071 Å for all non-hydrogen atoms.

## Supra­molecular Features   

**Figure 2:**
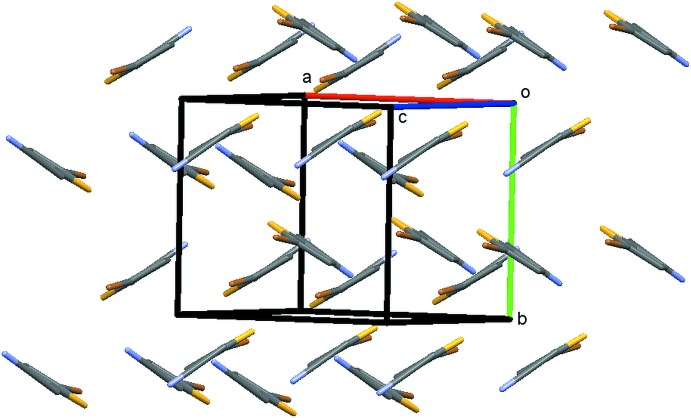
Packing diagram of **1** showing the herringbone packing pattern.

**Figure 3:**
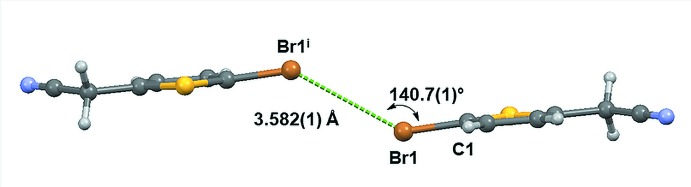
Inter­molecular halogen inter­action of **1**. Symmetry code: (i) 2 − *x*, −*y*, 1 − *z.*

**Figure 4:**
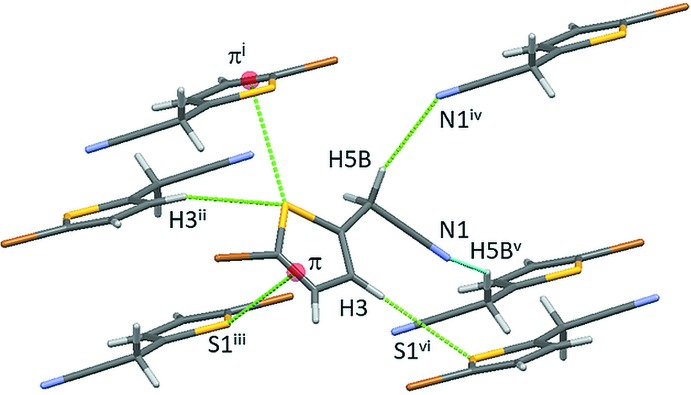
Inter­molecular inter­actions of **1**. Br⋯Br inter­actions omitted for clarity. π indicates the C1—C2 midpoint. Symmetry codes: (i) 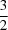 − *x*, −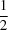 + *y*, 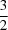 − *z*; (ii) −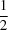 + *x*, 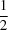 − *y*, −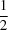 + *z;* (iii) 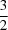 − *x*, 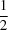 + *y*, 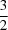 − *z;* (iv) 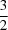 − *x*, −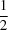 + *y*, 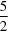 − *z;* (v) 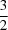 − *x*, 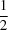 + *y*, 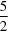 − *z*; (vi) 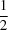 + *x*, 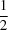 − *y*, 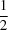 + *z.*

The structure packs with centrosymmetric π–π dimers, though the distance between least-squares planes formed by non-H atoms of the mol­ecules is beyond the sum of the van der Waals radii at 3.637 Å. Mol­ecules pack in a herringbone pattern with a dihedral angle of 65.2° between the least-squares planes formed by mol­ecules related by the 2_1_ screw axis (Fig. 2 ▸). The structure has several unique types of inter­molecular features. Each mol­ecule participates in centrosymmetric halogen-bonding dimers of Type I (Desiraju & Parthasarathy, 1989 ▸) with Br⋯Br contacts at 3.582 (1) Å and a C1—Br1⋯Br1 angle of 140.7 (1)° (Fig. 3 ▸). Each mol­ecule also engages in two weaker C—H⋯N inter­actions, one as an *sp* ^3^-hybridized C5—H5*B* donor and the other as an acceptor (N1) of this type of inter­action (Table 2 ▸, Fig. 4 ▸). It is noteworthy that the two methyl­ene hydrogen atoms are acidic on account of the electron-withdrawing nature of the cyano group and hence their participation in the formation of C—H⋯N hydrogen bonds is significant. Additionally, atom S1 contributes to two unique inter­molecular inter­actions. S1 acts as acceptor for an inter­action with C3—H3 as the donor. These S⋯H inter­actions are organized in a 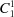
(4) graph-set motif parallel to [101]. An edge-to-face S1⋯π(C1—C2 midpoint) inter­action is also present at a distance of 3.391 Å (sum of van der Waals radii = 3.50 Å). These S⋯π close contacts are organized in chains parallel to [010].

## Hirshfeld surface analysis   

**Figure 5:**
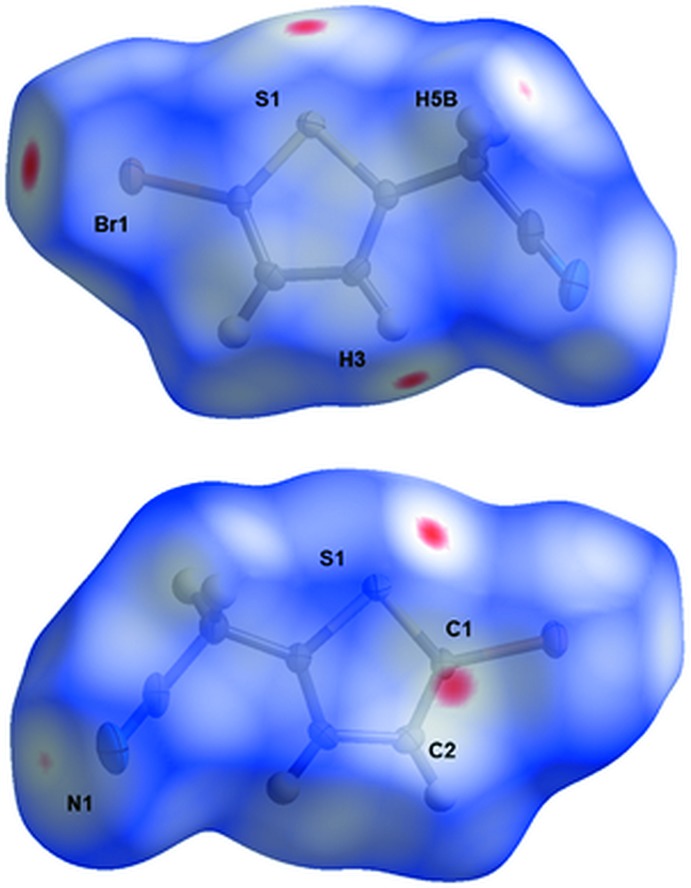
Hirshfeld surface of **1** mapped over *d*_norm_, shown in two orientations in the range −0.0639 to 0.93667 a.u. Red areas highlight inter­molecular contacts shorter than the sum of the van der Waals radii.

Inter­molecular inter­actions were studied further through analysis of the Hirshfeld surface, generated using *CrystalExplorer* (McKinnon *et al.* 2007 ▸; Spackman & Jayatilaka, 2009 ▸). Fig. 5 ▸ shows two orientations of the Hirshfeld surface mapped over *d*_norm_. The red areas of the surface indicate negative *d*_norm_ values corresponding to contacts closer than the sum of van der Waals radii and highlight the relevant inter­molecular inter­actions discussed. The relative surface-area contributions from the particular inter­atomic contacts described for **1** to the total Hirshfeld surfaces are summarized in Table 3 ▸. While N⋯H contacts comprise the largest percentage of contacts to the Hirshfeld surface described, the angular and distance components involved in the C—H⋯N hydrogen-bonding inter­actions do not suggest that these inter­actions dominate the packing. The Br⋯Br contacts comprise the smallest percentage of inter­atomic contacts described, however these Br⋯Br atom contacts [3.582 (1) Å] are the shortest of all the contacts described, relative to the van der Waals radii sums (−0.118 Å). The observation that C⋯C contacts comprise only a small percentage of the inter­atomic contacts is consistent with minor π–π stacking contributions and the observed stacking distance beyond the sum of the van der Waals radii.

## Database Survey   

A search of the current version of the Cambridge Structural Database (Version 5.39, updated November 2017; Groom *et al.*, 2016 ▸) yields a number of related structures with a 5-bromo­thio­phene fragment but only two non-salt structures with exclusively one small substituent in the 2-position. The structure of 2-acetyl-5-bromo­thio­phene (ACBTHO; Streur­man & Schenk, 1970 ▸) is planar like **1**, but the acetyl group is syn-periplanar relative to the sulfur of thio­phene, and Br⋯O=C inter­actions are present in the absence of Br⋯Br inter­actions. The structure of a co-crystal of 5-bromo­thio­phene-2-carb­oxy­lic acid with 5-fluoro­uracil (CAWCAP; Mohana *et al.*, 2017 ▸) is also similar, with no Br⋯Br inter­actions but the presence of Br⋯O=C inter­actions. No other structures of any substituted 2-thio­phene­aceto­nitrile have been reported.

The Type I Br⋯Br halogen-inter­action pattern of **1** is very similar to three other structures reported with only one bromine donor in the 5-position and no substitution in the 3- or 4-positions of the thio­phene group. The structures of 2-bromo-5-[4-(4-nitro­phen­yl)buta-1,3-dien-1-yl]thio­phene (MUJTUH; Kanibolotsky *et al.*, 2009 ▸), (2*E*)-1-(5-bromo-2-thien­yl)-3-(4-ethyl­phen­yl)prop-2-en-1-one (PUSKUL; Naik *et al.*, 2015 ▸), and (2*RS*,4*SR*)-2-exo-(5-bromo-2-thien­yl)-7-chloro-2,3,4,5-tetra­hydro-1*H*-1,4-ep­oxy-1-benzazepine (YUCTIA; Blanco *et al.*, 2009 ▸) have short inter­molecular Br⋯Br contacts with distances of 3.4619 (4), 3.4917 (5), and 3.5234 (7) Å, respectively, and centrosymmetric inter­actions with C—Br⋯Br angles of 145.12 (9), 151.37 (8), and 143.8 (1)°, respectively.

## Synthesis and Crystallization   

The title compound, 2-(5-bromo­thio­phen-2-yl)aceto­nitrile, was prepared according to the literature procedure (Lu *et al.*, 2014 ▸). Additional purification was performed by vacuum distillation (b.p. 334 K @ 0.07 mm Hg), which provided a colorless liquid that crystallized over several days to afford colorless crystals (m.p. 302–305 K) suitable for X-ray diffraction. EI–MS *m*/*z* (relative intensity) 202.88 (29.9), 200.89 (29.7), 123.02 (8.6), 122.01 (100.0), 95.03 (11.1).

## Refinement   

Crystal data, data collection and structure refinement details are summarized in Table 4 ▸. H atoms were placed in calculated positions and refined in the riding-model approximation with distances of C—H = 0.95 and 0.99 Å for the thio­phene and methyl­ene groups, respectively, and with *U*_iso_(H) = 1.2*U*_eq_(C).

A single low-angle reflection was rejected from these high-quality data sets due to the arrangement of the instrument with a conservatively sized beam stop and a fixed-position detector. The large number of reflections in the data sets (and the Fourier-transform relationship of intensities to atoms) ensures that no particular bias was thereby introduced.

## Supplementary Material

Crystal structure: contains datablock(s) global, I. DOI: 10.1107/S2056989018000968/dx2004sup1.cif

Structure factors: contains datablock(s) I. DOI: 10.1107/S2056989018000968/dx2004Isup2.hkl

CCDC reference: 1817195

Additional supporting information:  crystallographic information; 3D view; checkCIF report

## Figures and Tables

**Table 1:** Selected bond lengths (Å)

C1—C2	1.343 (6)	C4—C5	1.523 (7)
C2—C3	1.436 (6)	C5—C6	1.468 (7)
C3—C4	1.344 (7)		

**Table 2:** Hydrogen-bond geometry (Å, °)

*D*—H⋯*A*	*D*—H	H⋯*A*	*D*⋯*A*	*D*—H⋯*A*
C3—H3⋯S1^i^	0.95	2.93	3.844 (5)	162
C5—H5*B*⋯N1^ii^	0.99	2.66	3.425 (7)	134

Symmetry codes: (i) 
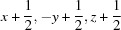
; (ii) 
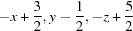
.

**Table 3:** Percentage contributions of inter­atomic contacts to the Hirshfeld surface

Contact	%
N⋯H/H⋯N	21.8
S⋯H/H⋯S	10.3
S⋯C/C⋯S	6.9
C⋯C	4.1
Br⋯Br	1.9

**Table 4:** Experimental details

Crystal data	
Chemical formula	C_6_H_4_BrNS
*M*_r_	202.07
Crystal system, space group	Monoclinic, *P*2_1_/*n*
Temperature (K)	173
*a*, *b*, *c* (Å)	9.775 (4), 7.278 (3), 10.698 (4)
β (°)	110.933 (8)
*V* (Å^3^)	710.8 (5)
*Z*	4
Radiation type	Mo *K*α
μ (mm^−1^)	6.00
Crystal size (mm)	0.51 × 0.44 × 0.22

Data collection	
Diffractometer	Rigaku XtaLAB mini
Absorption correction	Multi-scan (*REQAB*; Rigaku, 1998 ▸)
*T*_min_, *T*_max_	0.141, 0.267
No. of measured, independent and observed [*F* ^2^ > 2.0σ(*F* ^2^)] reflections	6585, 1444, 1198
*R*_int_	0.048
(sin θ/λ)_max_ (Å^−1^)	0.625

Refinement	
*R*[*F* ^2^ > 2σ(*F* ^2^)], *wR*(*F* ^2^), *S*	0.048, 0.117, 1.07
No. of reflections	1444
No. of parameters	82
H-atom treatment	H-atom parameters constrained
Δρ_max_, Δρ_min_ (e Å^−3^)	0.55, −0.82

Computer programs: *CrystalClear-SM Expert* (Rigaku, 2011 ▸), *SIR2004* (Burla *et al.*, 2005 ▸), *SHELXL2013* (Sheldrick, 2015 ▸), *Mercury* (Macrae *et al.*, 2008 ▸), *publCIF* (Westrip, 2010 ▸) and *CrystalStructure* (Rigaku, 2014 ▸).

## supplementary crystallographic information

### Crystal data

**Table:**

C_6_H_4_BrNS	*F*(000) = 392.00
*M**_r_* = 202.07	*D*_x_ = 1.888 Mg m^−^^3^
Monoclinic, *P*2_1_/*n*	Mo *K*α radiation, λ = 0.71075 Å
*a* = 9.775 (4) Å	Cell parameters from 5750 reflections
*b* = 7.278 (3) Å	θ = 3.5–26.5°
*c* = 10.698 (4) Å	µ = 6.00 mm^−^^1^
β = 110.933 (8)°	*T* = 173 K
*V* = 710.8 (5) Å^3^	Prism, colorless
*Z* = 4	0.51 × 0.44 × 0.22 mm

### Data collection

**Table:**

Rigaku XtaLAB mini diffractometer	1198 reflections with *F*^2^ > 2.0σ(*F*^2^)
Detector resolution: 6.849 pixels mm^-1^	*R*_int_ = 0.048
ω scans	θ_max_ = 26.4°, θ_min_ = 3.5°
Absorption correction: multi-scan (*REQAB*; Rigaku, 1998)	*h* = −12→12
*T*_min_ = 0.141, *T*_max_ = 0.267	*k* = −9→9
6585 measured reflections	*l* = −13→13
1444 independent reflections	

### Refinement

**Table:**

Refinement on *F*^2^	Secondary atom site location: difference Fourier map
*R*[*F*^2^ > 2σ(*F*^2^)] = 0.048	Hydrogen site location: inferred from neighbouring sites
*wR*(*F*^2^) = 0.117	H-atom parameters constrained
*S* = 1.07	*w* = 1/[σ^2^(*F*_o_^2^) + (0.0495*P*)^2^] where *P* = (*F*_o_^2^ + 2*F*_c_^2^)/3
1444 reflections	(Δ/σ)_max_ < 0.001
82 parameters	Δρ_max_ = 0.55 e Å^−^^3^
0 restraints	Δρ_min_ = −0.82 e Å^−^^3^
Primary atom site location: structure-invariant direct methods	

### Special details

**Table:**

Geometry. All esds (except the esd in the dihedral angle between two l.s. planes) are estimated using the full covariance matrix. The cell esds are taken into account individually in the estimation of esds in distances, angles and torsion angles; correlations between esds in cell parameters are only used when they are defined by crystal symmetry. An approximate (isotropic) treatment of cell esds is used for estimating esds involving l.s. planes.
Refinement. Refinement was performed using all reflections. The weighted R-factor (wR) and goodness of fit (S) are based on F^2^. R-factor (gt) are based on F. The threshold expression of F^2^ > 2.0 sigma(F^2^) is used only for calculating R-factor (gt).

### Fractional atomic coordinates and isotropic or equivalent isotropic displacement parameters (Å^2^)

**Table:**

	*x*	*y*	*z*	*U*_iso_*/*U*_eq_	
Br1	0.90954 (6)	0.12818 (8)	0.59072 (5)	0.0348 (2)	
S1	0.76060 (13)	0.07396 (16)	0.79952 (11)	0.0242 (3)	
N1	0.8679 (6)	0.3162 (8)	1.2745 (5)	0.0610 (16)	
C1	0.9070 (5)	0.1545 (6)	0.7644 (4)	0.0218 (10)	
C2	1.0043 (5)	0.2468 (6)	0.8663 (4)	0.0258 (11)	
H2	1.0914	0.3016	0.8634	0.031*	
C3	0.9592 (5)	0.2517 (6)	0.9801 (5)	0.0261 (11)	
H3	1.0143	0.3093	1.0624	0.031*	
C4	0.8308 (5)	0.1662 (6)	0.9581 (4)	0.0220 (10)	
C5	0.7453 (6)	0.1396 (6)	1.0510 (5)	0.0314 (12)	
H5A	0.6437	0.1841	1.0063	0.038*	
H5B	0.7410	0.0071	1.0701	0.038*	
C6	0.8137 (6)	0.2395 (7)	1.1774 (5)	0.0382 (14)	

### Atomic displacement parameters (Å^2^)

**Table:**

	*U*^11^	*U*^22^	*U*^33^	*U*^12^	*U*^13^	*U*^23^
Br1	0.0342 (4)	0.0494 (4)	0.0253 (3)	−0.0001 (2)	0.0160 (3)	−0.0028 (2)
S1	0.0219 (7)	0.0271 (6)	0.0233 (6)	−0.0037 (5)	0.0077 (5)	−0.0011 (5)
N1	0.079 (5)	0.077 (4)	0.038 (3)	0.001 (3)	0.034 (3)	−0.012 (3)
C1	0.023 (3)	0.025 (2)	0.019 (2)	0.0048 (19)	0.0088 (19)	0.0027 (17)
C2	0.016 (3)	0.029 (3)	0.032 (3)	0.0024 (19)	0.008 (2)	0.0032 (19)
C3	0.026 (3)	0.027 (3)	0.021 (2)	0.001 (2)	0.005 (2)	−0.0052 (18)
C4	0.023 (3)	0.024 (2)	0.021 (2)	0.0026 (19)	0.009 (2)	0.0022 (18)
C5	0.034 (3)	0.036 (3)	0.027 (3)	0.000 (2)	0.013 (2)	0.002 (2)
C6	0.044 (4)	0.049 (4)	0.028 (3)	0.011 (3)	0.021 (3)	0.006 (2)

### Geometric parameters (Å, º)

**Table:**

Br1—C1	1.877 (4)	C3—C4	1.344 (7)
S1—C1	1.708 (5)	C3—H3	0.9500
S1—C4	1.723 (4)	C4—C5	1.523 (7)
N1—C6	1.131 (7)	C5—C6	1.468 (7)
C1—C2	1.343 (6)	C5—H5A	0.9900
C2—C3	1.436 (6)	C5—H5B	0.9900
C2—H2	0.9500		
			
C1—S1—C4	90.7 (2)	C3—C4—C5	129.8 (4)
C2—C1—S1	113.5 (4)	C3—C4—S1	111.8 (3)
C2—C1—Br1	126.8 (4)	C5—C4—S1	118.4 (3)
S1—C1—Br1	119.4 (3)	C6—C5—C4	111.2 (4)
C1—C2—C3	110.9 (4)	C6—C5—H5A	109.4
C1—C2—H2	124.5	C4—C5—H5A	109.4
C3—C2—H2	124.5	C6—C5—H5B	109.4
C4—C3—C2	113.0 (4)	C4—C5—H5B	109.4
C4—C3—H3	123.5	H5A—C5—H5B	108.0
C2—C3—H3	123.5	N1—C6—C5	179.2 (6)
			
C4—S1—C1—C2	−0.1 (4)	C2—C3—C4—S1	0.8 (5)
C4—S1—C1—Br1	−174.5 (3)	C1—S1—C4—C3	−0.4 (4)
S1—C1—C2—C3	0.6 (5)	C1—S1—C4—C5	−179.6 (4)
Br1—C1—C2—C3	174.5 (3)	C3—C4—C5—C6	5.7 (7)
C1—C2—C3—C4	−0.9 (6)	S1—C4—C5—C6	−175.2 (3)
C2—C3—C4—C5	179.8 (4)		

### Hydrogen-bond geometry (Å, º)

**Table:**

*D*—H···*A*	*D*—H	H···*A*	*D*···*A*	*D*—H···*A*
C3—H3···S1^i^	0.95	2.93	3.844 (5)	162
C5—H5*B*···N1^ii^	0.99	2.66	3.425 (7)	134

Symmetry codes: (i) *x*+1/2, −*y*+1/2, *z*+1/2; (ii) −*x*+3/2, *y*−1/2, −*z*+5/2.
